# Ensemble Nonlinear Autoregressive Exogenous Artificial Neural Networks for Short-Term Wind Speed and Power Forecasting

**DOI:** 10.1155/2014/972580

**Published:** 2014-09-08

**Authors:** Zhongxian Men, Eugene Yee, Fue-Sang Lien, Zhiling Yang, Yongqian Liu

**Affiliations:** ^1^Waterloo CFD Engineering Consulting Inc., Waterloo, ON, Canada N2T 2N7; ^2^Department of Mechanical & Mechatronics Engineering, University of Waterloo, Waterloo, ON, Canada N2L 3G1; ^3^Defence Research and Development Canada, Suffield Research Centre, P.O. Box 4000, Stn Main, Medicine Hat, AB, Canada T1A 8K6; ^4^School of Renewable Energy, North China Electric Power University, Beijing 102206, China

## Abstract

Short-term wind speed and wind power forecasts (for a 72 h period) are obtained using a nonlinear autoregressive exogenous artificial neural network (ANN) methodology which incorporates either numerical weather prediction or high-resolution computational fluid dynamics wind field information as an exogenous input. An ensemble approach is used to combine the predictions from many candidate ANNs in order to provide improved forecasts for wind speed and power, along with the associated uncertainties in these forecasts. More specifically, the ensemble ANN is used to quantify the uncertainties arising from the network weight initialization and from the unknown structure of the ANN. All members forming the ensemble of neural networks were trained using an efficient particle swarm optimization algorithm. The results of the proposed methodology are validated using wind speed and wind power data obtained from an operational wind farm located in Northern China. The assessment demonstrates that this methodology for wind speed and power forecasting generally provides an improvement in predictive skills when compared to the practice of using an “optimal” weight vector from a single ANN while providing additional information in the form of prediction uncertainty bounds.

## 1. Introduction

With recent rapid advances in the development of clean energy, wind power has increasingly become an important component of a renewable and sustainable energy system. Even so, the problem of the variability of wind power production is well known, arising as such from unpredictable fluctuations in the wind speed and direction on short timescales and from seasonal variations on longer timescales. In consequence, the variability in wind power generation needs to be properly managed in order to facilitate the optimal integration of this form of renewable energy into the electrical grid system. The efficient power system management of an electrical grid system that includes wind energy generation in its energy production portfolio requires necessarily the capability to forecast wind power generation. Historical studies provide evidence that the power generated by a wind turbine is related primarily to the wind velocity that occurs at or near the location of the wind turbine. In this paper, we examine the predictability of the wind speed and wind turbine power (as well as the correlation between these two quantities) using an artificial neural network methodology. In other words, we are interested in modeling the structural dependence between wind speed and wind power and the predictability of one from the other as well as the predictability of the wind speed itself (which is the critical quantity that determines wind power production). More specifically, we focus on short-term wind energy forecasting (up to 72 hours) which is critical for managing wind power production and for energy trading. To this purpose, we demonstrate the utility of artificial neural networks for providing short-term forecasts of wind speed and of the associated wind turbine power.

Over the past two decades, modeling and forecasting wind speed and wind power have become an intensive research area. Generally speaking, there are three methodologies that have been used for wind speed and wind power prediction. The first methodology is based on statistical models that characterize the correlation structure in measured wind speed and wind power time series (historical data obtained at a wind farm site). Time series analysis models such as the autoregressive (AR) models, moving average (MA) models, and autoregressive integrated moving average (ARIMA) models have been applied to these types of historical data. Erdem and Shi [1] and Torres et al. [2] used an ARIMA model for forecasting the wind speed and direction. More recently, financial time series models such as the autoregressive conditional heteroskedasticity (ARCH) model of Engle [3] and the generalized autoregressive conditional heteroskedasticity (GARCH) model of Bollerslev [4] have been applied to model the variability in temporal fluctuations of the wind speed. This approach models the residuals in the time series using the more sophisticated GARCH model, rather than simply treating these residuals as (uncorrelated) white noise. In particular, the ARIMA-GARCH and the GARCH-in-mean (GARCH-M) models proposed by Liu et al. [5] have been used to model the mean and variability in measured wind speed time series. One of the advantages in using these models is that the algorithms and the corresponding software packages that implement these algorithms are readily available in the open literature and can be applied to estimate the parameters of these complicated models. The disadvantage is that these models use only a single historical time series of the wind speed variations for the analysis and subsequent forecasting. This, to some extent, is not realistic and the results of the analysis may not be satisfactory for real applications (e.g., short-term energy forecasting).

The second methodology consists of using a physics-based (weather) forecasting model for wind speed prediction, which in turn can be used for wind power prediction. These physics-based numerical weather prediction models solve the governing equations for fluid flow supplemented by relevant subgrid scale parameterization schemes for various atmospheric processes that cannot be properly resolved in the simulation. Indeed, improvements in weather forecasting (through increased model resolution which places less burden on the correct formulation of subgrid scale parameterization schemes) are limited by the available computational resources. Li et al. [6] introduced a short-term wind speed forecasting technique based on precalculated flow fields obtained using computational fluid dynamics (CFD).

The third methodology for wind speed forecasting utilizes machine learning approaches. Within this class of methods, artificial neural network (ANN), fuzzy systems theory, and other related paradigms such as grey predictors and support vector machine (SVM) have been applied. Damousis and Dokopoulos [7], Damousis et al. [8], and Pinson and Kariniotakis [9] used fuzzy model expert systems for wind speed and power forecasting, whereby the relationship between the independent and dependent variables is described by a fuzzy mapping system. Before the fuzzy system can be used for wind speed and wind power forecasting, the system has to “learn” the mapping from the training data. Zeng and Qiao [10] and Mohandes et al. [11] used SVM methods for short-term wind power and wind speed prediction. The SVM has been applied successfully for pattern recognition, for instance, in data classification. The idea behind the SVM is to first project the covariates from a lower-dimensional to a higher-dimensional space. The dependent variable is then projected into this higher-dimensional space, leading to a nonlinear regression from the original lower-dimensional to the higher-dimension space. The parameters in a SVM can be obtained by solving a dual quadratic programming problem. Artificial neural networks have been also widely applied to characterize the relationship between wind power and the related wind speed. An ANN is constructed from layers of interconnected nodes, each of which processes the information it receives and passes this along to other nodes in the network through weighed connections. An overview of the application of the above three methodologies for wind speed forecasting can be found in Foley et al. [12].

Unlike the more conventional statistical and physics-based approaches for wind speed forecasting, machine learning approaches do not explicitly formulate a specific parametric model for the process but instead base the analysis on the use of a “black box" or “grey box" formulation of the problem. These black or grey boxes representing the wind speed processes are then “trained” using historical data sets. Once the performance of the trained neural network, fuzzy system, or support vector machine is tested in accordance with some statistical criteria, these essentially “nonparametric” models for the process can be used to predict (forecast) the wind speed and wind power.

It is well known that the problem of one-step-ahead forecasting is relatively easy when compared to the related problem of multi-step-ahead forecasting. For this purpose, it has generally been found that forecasting based on the information obtained from a single time series is not very accurate. For this reason, wind speed and wind power forecasting are usually performed using some covariate (or independent) random variables. In the literature, numerical weather prediction (NWP) data for wind speed are often used as the covariate variable when multi-step-ahead wind power prediction is made. Because NWP data usually provide adequate predictions of various meteorological variables, wind speed and wind power predictions at a wind turbine using NWP data as the covariate variable should (as a general rule) lead to more reliable predictions of wind speed and power as compared to the predictions of these quantities based on historical data alone. Yang and Liu [13] used feedforward back-propagation ANNs based on NWP data for forecasting wind speed and the concomitant power of a wind turbine.

Recently, model averaging ANN approaches have been developed and used for wind speed and wind power forecasting as, for example, by Han et al. [14], Li et al. [15], and Wang et al. [16]. In the literature, there are principally two methods that have been used for model averaging in the context of ANN. One method uses a simple averaging approach, whereas the other uses a weighted averaging approach in which the weights are estimated by solving a constrained optimization problem. It is well known that model averaging can reduce the prediction errors and, consequently, improve the forecasting accuracy. Because a large sample of ANN realizations is usually obtained for model averaging, the confidence intervals for the predicted values can be easily calculated. An attractive alternative approach to using simple or weighted averaging is to apply a Bayesian methodology and average the outputs of many ANN realizations having weights sampled from a posterior distribution (giving essentially a probability weighting). In this context, Blonbou [17] proposed applying an ANN trained using an adaptive Bayesian learning method for very short-term wind power forecasting.

In this paper, we propose a new methodology for forecasting wind speed and wind turbine power. The approach uses a numerical weather prediction model to specify the boundary conditions for a high-resolution CFD model to make predictions of the wind flow field at the microscale (which are made available in the form of a wind field library). The wind flow information is then used as the exogenous input to an ensemble of nonlinear autoregressive ANNs that are trained and subsequently used for prediction (forecasting) of the wind speed at turbine hub height and the wind power generated by the turbine. A simple modification of this approach involves applying a bilinear interpolation to available NWP data and using this piece of information as the exogenous input for an ensemble of ANNs. In the latter case, the multi-step-ahead wind speed and wind power prediction is based on the extracted wind speed data from an NWP database. For wind power forecasting, the measurements of wind power were censored above at a certain maximum level. In consequence, it is not correct to map an unconstrained wind speed time series to a censored wind power time series. In view of this fact, we demonstrate the performance of an ANN that accounts properly for the censoring of the wind power for use in forecasting.

This paper is organized as follows.  Section 2 introduces the general architecture of ANNs in terms of the activation function and the methodology used for network training.  Section 3 presents multi-step-ahead wind speed and wind power forecasting, compares these forecasts to the actual measured values, and conducts an assessment of the prediction efficacy using two different criteria.  Section 4 conducts an assessment of the forecasting efficacy for the censored wind power data, while Section 5 provides the conclusions of the paper.

## 2. Architecture of ANNs

### 2.1. Background

Let *x*_*t*_ denote a measurement of the wind speed at or near a wind turbine at time *t* (output sample value) with *t* ≤ *T*, where *T* is a positive integer corresponding to the sample size. Similarly, let *y*_*t*_ denote a measured wind power at the corresponding wind turbine at time *t* (output sample value) with *t* ≤ *T*. One goal of this paper is to predict the wind speed over a fixed period after time *T*, say from *T* + 1 to *T* + *d*, where *d* is a positive integer. The second goal is to forecast the wind power generated by the corresponding wind turbine, also over the period from *T* + 1 to *T* + *d*. As we reviewed earlier, one strategy for performing these two tasks is to predict the wind speed and wind power, respectively, based on the previously observed data, while the other strategy is to use the NWP or CFD wind speed data as an exogenous input for the prediction of the wind speed and wind power at some future time. A third option is to utilize both current and previously observed data and exogenous inputs for the forecasting of the wind speed and power. To this end, suppose that the sampled precalculated wind speed, obtained either from the computational fluid dynamics model or from a bilinear interpolation of the NWP data, is denoted by *z*_*t*_ with *t* = 1,…, *T* (exogenous input). Then the wind speed and wind power at time *t* can be modeled, respectively, as the following nonlinear autoregressive exogenous process:

(1) xt=f(xt−1,xt−2,…,xt−p,zt,zt−1,…,zt−q)+ϵt,aaaaaaaaaaaaaaaaaaaaaaaaaaat=1,2,…,T

(2) yt=g(yt−1,yt−2,…,yt−p,zt,zt−1,…,zt−q)+et,aaaaaaaaaaaaaaaaaaaaaaaaaaat=1,2,…,T,

where **ϵ** = (*ϵ*_1_,…,*ϵ*_*T*_)′ and **e** = (*e*_1_,…,*e*_*T*_)′ are independent “noise” vectors with ′ denoting matrix transposition (namely, the components of these vectors are the model errors in the prediction models for *x*_*t*_ and *y*_*t*_ for *t* = 1,2,…, *T*, resp.). The components of each of these noise vectors are assumed to have zero mean and constant variance. The nonlinear mapping functions *f*(·) and *g*(·) are used to represent the complex relationship that exists between the current value of the wind speed or wind power and previous values of these quantities and the exogenous inputs. The order of the model is represented by the nonnegative integers *p* and *q*, and with the specification of these two parameters we can see that the model embodied in (1) and (2) has the form of a nonlinear autoregression model in which the wind speed *x*_*t*_ or wind power *y*_*t*_ is regressed on their respective previous values (up to order *p*) and on the current and previous values of the exogenous input (up to order *q*). This model structure when used in conjunction with an artificial neural network for representation of the (unknown) functional forms for *f*(·) and *g*(·) will be referred to as a nonlinear autoregressive exogenous (NARX) ANN (or, more specifically, as a NARX(*p*, *q*) ANN when the model orders *p* and *q* for the dependent or output variables and for the exogenous variables, resp., are explicitly given). As a simple convention, a NARX model with *p* = 0 will imply a model in which the output (response) variable is not assumed to have any dependence on its previous value(s) (namely, the response variable is assumed to depend only on the current and previous values of the exogenous input). Similarly, a NARX model with *p* = 0 and *q* = 0 will imply a model in which the output variable at a given time depends only on the current value of the exogenous variable at the same time.

The functions *f*(·) and *g*(·) in (1) and (2) are two nonlinear mapping functions that are chosen to characterize properly the complicated relationships between *x*_*t*_ and *y*_*t*_ and their respective previous values and the current and previous values of the exogenous input *z*_*t*_. In the ANN formulation, each of these two relationships can be modeled by using a multilayer neural network system in which each layer consists of many neurons (or perceptron nodes). The input of each node in a given layer of the neural network is a linear combination of the outputs of the nodes in the preceding layer plus a bias. A function that transforms the input to the output of a node is called a transfer function or an activation function. As demonstrated by Hornik et al. [18] and White [19], subject to some regularity conditions, a three-layer neural network consisting of an input layer, an output layer, and a hidden layer can approximate arbitrarily well any continuous function of multivariate real variables, given that the number of nodes in the hidden layer is sufficiently large. In consequence of this “universal approximation theorem”, we focus in this paper on three-layer neural network structures with a variable number of nodes in the hidden layer.

The input layer acts as a buffer to “feed” the input signals *ζ* into the hidden layer (where *ζ* ≡ *x* or *y* (previous values) and/or *z* (present and previous values) in (1) and (2), resp.). Each node in the hidden layer has an input which sums up the input signals *ζ*_*i*_ through weighted connections *w*_*ki*_^*h*^, with the output *v*_*k*_^*h*^ of the *k*th node in the hidden layer given by

(3) $$\begin{array}{c} v_{k}^{h}=h\left(\underset{i}{\sum }w_{ki}^{h}\zeta_{i}+b_{k}^{h}\right),\;k=1,\ldots ,\tilde{q}, \end{array}$$

where *b*_*k*_^*h*^ is the bias for node *k*, $\tilde{q}$ is the number of nodes in the hidden layer, and *h* is an activation function for the hidden layer.

Similarly, the output of nodes in the output layer is given as follows:

(4) $$\begin{array}{c} v_{l}^{o}=o\left(\underset{k}{\sum }w_{lk}^{o}v_{k}^{h}+b_{l}^{o}\right),\;l=1,\ldots ,\tilde{r}, \end{array}$$

where *o* is the activation function for the output layer, *w*_*lk*_^*o*^ and *b*_*l*_^*o*^ are the weights and bias, respectively, for the *l*th node of the output layer, $\tilde{r}$ is the number of nodes in the output layer, and *v*_*l*_^*o*^ is the output of the *l*th node of the output layer. The activation functions must be piecewise continuous for our purposes and can be chosen to be a simple linear function, a sigmoidal function, a hyperbolic tangent function, or a radial basis function (see Table 1). More specifically, as demonstrated by Hornik et al. [18], a “universal approximation theorem” states that any continuous function can be approximated with a neural network having at least three layers provided that the activation function *h*(*x*) is locally bounded and piecewise continuous. Aside from these conditions, the activation function for the hidden layer is arbitrary. In view of these conditions, we choose the logistic sigmoidal function as the activation function for the hidden layer (namely, we choose *h*(*x*) = 1/(1 + exp⁡(−*x*))). Usually, the activation function for the output layer is simply a linear function, so we use *o*(*x*) = *x*. For more information on the structure of ANNs and their myriad applications, the reader is referred to Kermanshahi [20] and Yuen and Lam [21].

For our problem, the outputs of the output layer of the neural network are identified as either *x* (wind speed) or *y* (wind power). Let us generically label the outputs as *ϕ* (*ϕ* ≡ *x*, *y*) and define the output vector of an ANN by $\hat{\phi }={\left({\hat{\phi }}_{1},\ldots ,{\hat{\phi }}_{T}\right)}^{\prime }$ (namely, we identify *v*_*l*_^*o*^ with ${\hat{\phi }}_{l}$ in (4)). Before the neural network can be used for prediction, it is necessary to first train the network. The training process is intended to minimize an objective function, which we take to be either the root-mean-square error (RMSE) function or the mean absolute error (MAE) function between the expected output $\hat{\phi }$ and the measured (observed) output *ϕ* (which constitute the training set):

(5) $$\begin{array}{c} \text{RMSE}={\left(\frac{1}{T}\overset{T}{\underset{i=1}{\sum }}{\left(\phi_{i}-{\hat{\phi }}_{i}\right)}^{2}\right)}^{1/2}, \end{array}$$

or

(6) $$\begin{array}{c} \text{MAE}=\frac{1}{T}\overset{T}{\underset{i=1}{\sum }}\left|\phi_{i}-{\hat{\phi }}_{i}\right|. \end{array}$$

### 2.2. Neural Network Training

As mentioned earlier, there are several optimization algorithms that can be utilized for training an ANN. For this purpose, the weights and biases that define the multilayer ANN will collectively be called the network parameter vector ***θ***. We wish to choose the network parameters ***θ*** so as to minimize either the RMSE or MAE on the given training set. It is noted that these objective functions are highly nonlinear, multimodal functions of the network parameter vector, whose minimization poses a significantly difficult problem. Traditionally, the conjugate gradient or Levenberg-Marquardt and particle swarm optimization (PSO) methods have been used for network training. The principal difference between these two methods is that the former is a local optimization method, while the latter is a global optimization method. Given the highly multimodal nature of the objective function to be minimized, the PSO method generally tends to provide a “better” solution for the network parameter values (weights) in the sense of providing a better fitting model with a lower misfit value. The PSO algorithm was originally proposed by Kennedy and Eberhart [22, 23] and is based on studies of the behaviour of animal movements such as in the flocking of birds, the schooling of fish, and more generally the theory of swarm intelligence. We found that the application of the PSO method to the problem of network training allowed for fast efficient training of moderately large network structures, with computational speeds that are comparable to or better than that obtained using the Levenberg-Marquardt method.

Once a neural network has been properly trained, it can be used to make forecasts of the mean wind speed *x*_*t*_ and the wind power *y*_*t*_, for *t* = *T* + 1, *T* + 2,…, *T* + *d*. In this paper, we assume that *d* = 72 because we are interested in evaluating the efficacy of the proposed methodology for short-term wind speed and power forecasting (up to 72 h).

### 2.3. An Ensemble ANN Model

It has been observed that multiple ANN models can provide the same or comparable fits to a given data set used to train the network. The principal difficulty in the application of the ANN framework resides in the determination of the number of nodes (or, equivalently, weights and biases) to use in the representation of the neural network. In this regard, finding a single (so-called) optimal network parameter vector in the training process can lead to overfitting and subsequently to poor generalization by the network resulting in poor predictive performance. It is a challenging problem to assess whether any single trained ANN is suitable for prediction but, nevertheless, it is standard practice to apply conventional network training to determine a single optimal network and use this network for forecasting, even though this single network may not be able to provide an accurate prediction for output values.

In principle, an ANN that fits a training data set well does not imply necessarily that it is also appropriate for the provision of an out-of-sample prediction. In other words, the accuracy of an out-of-sample prediction may not depend on how well a particular ANN model can fit the training data. Sometimes, a selected ANN may overfit the training data and, as a consequence, provides poor generalization vis-à-vis the provision of an out-of-sample prediction. To address this problem, we consider the use of multiple ANNs which are trained to fit the given data set. This leads to an ensemble-based ANN framework which involves generating multiple possible realizations of ANNs which fit the training data by considering a suite of ANNs with different numbers of hidden layer nodes, each of which is trained by setting a number of different random values for the weights (which in principle can be sampled from a Gaussian distribution). In this manner, an ensemble of competing ANN models can be identified each of which is representative of the given training data set.

When multiple ANNs are trained and used for forecasting, one must determine how to combine each network's prediction to give an aggregate forecast. For this purpose, we use a weighted averaging method to combine all component models (in the ensemble) in order to conduct an out-of-sample multi-step-ahead forecasting of the wind speed and power. More specifically, the *i*th component model (in the ensemble) is assigned a weight *w*_*i*_ ∈ [0,1]. These weights are determined by solving a quadratic programming problem. As an example (cf. (1)), suppose that there are *N* members in the ensemble and let $\left\{{\tilde{x}}^{1},\ldots ,{\tilde{x}}^{N}\right\}$ be the set of predictions of the observation vector **x** (wind speed). Let $\tilde{X}\equiv ({\tilde{x}}^{1},\ldots ,{\tilde{x}}^{N})$ denote the corresponding prediction matrix. Then, we can form the set of residual vectors $\left\{{\tilde{e}}^{1},\ldots ,{\tilde{e}}^{N}\right\}$ with ${\tilde{e}}^{i}\equiv x-{\tilde{x}}^{i}$ (*i* = 1,…, *N*). The corresponding residual matrix is defined as $\tilde{E}=({\tilde{e}}^{1},\ldots ,{\tilde{e}}^{N})$. Let us denote by **w** = (*w*_1_,…,*w*_*N*_)′ the weight vector associated with the component ANNs in the ensemble (and these weights should not be confused with the weights that define an individual neural network). Naturally, the components of the weight vector **w** are nonnegative with ∑_*i*=1_^*N*^ *w*_*i*_ = 1. The ensemble residual vector is the weighted sum of the component residuals of the ensemble defined as $\tilde{E}(w)\equiv \tilde{E}w$. The weights can be determined by minimizing the following objective function:

(7) $$\begin{array}{c} {ɛ}_{N}\left(w\right)=\frac{1}{N}{||\tilde{E}\left(w\right)||}^{2}=w^{\prime }H_{N}w, \end{array}$$

where ||·|| represents the Euclidean norm and $H_{N}\equiv (1/N){\tilde{E}}^{\prime }\tilde{E}$ is an (*N* × *N*) matrix.

Once the weights for the ensemble ANNs have been obtained by solving the quadratic programming problem described above, we can use them to make multi-step-ahead predictions. Define by $\left\{{\hat{x}}^{1},\ldots ,{\hat{x}}^{N}\right\}$ the set of multi-step-ahead prediction vectors for the “future” observations of (*x*_*T*+1_,…,*x*_*T*+*d*_)′. Then the ensemble prediction of the multi-step-ahead measurements of *x*_*t*_ is

(8) $$\begin{array}{c} {\hat{x}}_{t}\left(\hat{w}\right)=\overset{N}{\underset{i=1}{\sum }}{\hat{w}}_{i}{\hat{x}}_{t}^{i},\;t=T+1,\ldots ,T+d, \end{array}$$

where $\hat{w}$ denotes the solution to the minimization of *ε*_*N*_(**w**) given in (7).

Finally, another advantage of using an ensemble of ANNs for prediction is that the information embodied in the ensemble can be used to determine confidence intervals for the forecast. To calculate the confidence intervals in the forecast, we assume that the samples in the set $\left\{{\hat{x}}^{i},i=1,\ldots ,N\right\}$ are random and use the following formula for the estimate of *x*_*t*_ (namely, for (*x*_*t*_)_est_):

(9) $$\begin{array}{c} {\left(x_{t}\right)}_{\text{est}}={\hat{x}}_{t}\left(\hat{w}\right)\pm {\hat{\sigma }}_{t}t_{\alpha ,v},\;t=T+1,\ldots ,T+d, \end{array}$$

with *t*_*α*,*v*_ being the *α*-level critical value of a Student's *t*-distribution with *v* = *N* − 1 degrees of freedom. The standard sample error vector $\hat{\sigma }={\left({\hat{\sigma }}_{T+1},\ldots ,{\hat{\sigma }}_{T+d}\right)}^{\prime }$ is defined as

(10) $$\begin{array}{c} {\hat{\sigma }}_{t}={\left(\overset{N}{\underset{i=1}{\sum }}{\hat{w}}_{i}{\left[{\hat{x}}_{t}^{i}-{\bar{x}}_{t}\right]}^{2}\right)}^{1/2},\;t=T+1,\ldots ,T+d, \end{array}$$

where ${\bar{x}}_{t}=\sum_{i=1}^{N}{\hat{x}}_{t}^{i}/N$ is the (ensemble) mean of the samples in the set $\left\{{\hat{x}}_{t}^{1},\ldots ,{\hat{x}}_{t}^{N}\right\}$. Because this is a biased estimator of the standard deviation, we use instead the following formula:

(11) σ^t=((1−∑n=1Nw^n2)−1·∑i=1Nw^i[x^ti−x¯t]2)1/2,aaaaaaaaaaaaaaaaaaat=T+1,…,T+d,

to obtain an unbiased estimator of the standard deviation.

## 3. Empirical Analysis of Wind Speed and Wind Power Forecasting

### 3.1. Historical Wind Speed and Power Data Preparation

We have two data sets of wind speed and power measured in the vicinity of a wind turbine (designated as WT24 for wind turbine number 24) forming part of a large wind farm in Northern China. More specifically, one data set consists of hourly-averaged wind speeds at WT24, and the other data set consists of the corresponding hourly-averaged power generated by the same wind turbine. The problem to be addressed is to provide a forecast for the future wind speed and power at this turbine.

Rather than forecasting multi-step-ahead wind speeds and wind power using ANNs trained solely with historical data (time series) for these quantities, we will use model wind speed data at WT24 extracted from either numerical weather prediction or computational fluid dynamics modeling in addition to the historical data. This model wind speed data will be used as the exogenous covariate information for neural network training and forecasting. For this purpose, the model wind speed was extracted from either an NWP database or an extensive terrain-resolving wind field library for the spatial domain occupied by the wind farm. A simple bilinear interpolation (BI) was applied to the coarse-resolution NWP wind speed data to obtain the required wind speed at the location of the WT24 wind turbine (namely, the wind speed was interpolated from the four available points forming the corners of the grid square in which WT24 lies).

The objective is the forecasting of the wind speed and power at WT24, and towards that purpose we train an ensemble of neural networks using historical time series data of measured wind speed and power at WT24 and model wind speed obtained from either a CFD wind field library or a BI of NWP wind data (the latter of which forms the exogenous input to the network). In the following analysis, we apply a weighted averaging operation (as described in the previous section) to combine all individual ANN predictions obtained from the ensemble members in order to provide the multi-step-ahead wind speed and power forecasting at WT24 for a 72-hour time period. In this paper, the term “multi-step-ahead forecast” is assumed to be synonymous with “short-term forecast” (namely, by which a forecast of the wind speed or power for a short period (up to several days) in the future is meant). The historical wind speed and power data were collected hourly over a period of 18 days at WT24. In the ensuing analysis, the first 15 days of wind speed and power data was used for neural network training and the last 3 days of this data was reserved for the assessment of the efficacy of the multi-step-ahead forecasting of these quantities using the ensemble-based ANN framework. We will compare the wind speed and power forecast efficacy (at WT24) for two different NARX ANN models, namely, (1) a NARX(0,0) ANN model (namely, *p* = *q* = 0 in which the dependent variable at a given time depends only on the current value of the exogenous input) and (2) the optimal NARX(*p*, *q*) ANN model with *p* ∈ {1,2} and *q* ∈ {1,2} allowing for the modeling of a possible temporal correlation structure in the previous dependent and current and previous exogenous variables used for the wind speed and power forecasting. In each NARX ANN model, the exogenous inputs correspond to either NWP (interpolated) or CFD wind data.

### 3.2. Numerical Weather Prediction Wind Data

The NWP wind data was obtained from a mesoscale model which used numerical computational methods to solve the governing fluid mechanics and thermodynamics equations for describing the weather evolution process starting from some initial conditions. This model was used to predict the future evolution of the state of the atmosphere (wind velocity, temperature, humidity, etc.). For this purpose, the Weather Research and Forecasting Model (WRF) was used. This model is a mesoscale NWP system developed by the National Center for Atmospheric Research (NCAR), the National Center for Environmental Prediction (NCEP), and other research institutions in USA to support both atmospheric research and operational forecasting needs [24]. The NWP wind data used in the paper was obtained from WRF, with the initial field and boundary conditions required by WRF obtained from reanalysis data published by NCEP. This model data was archived in an NWP database and consisted of the wind speed and direction at the hub height of the wind turbine. The NWP wind speed and direction data were obtained at a temporal resolution of 1 h on a computational grid centered on the location of the wind farm with a spatial resolution of 3 km.

### 3.3. Computational Fluid Dynamics Wind Data

The CFD model used in the present study is part of the urban (microscale) flow modeling system developed at Waterloo CFD Engineering Consulting Inc. and Defence R&D Canada, Suffield Research Centre. This system has been used extensively for modeling the dispersion of contaminants in urban environments [25–27]. This modeling system is fully described by Yee et al. [28] and, as a consequence, only a very brief overview of the system pertinent to the present study will be given here.

The module urbanGRID was used to automatically generate a body-fitted (or curvilinear) grid to accommodate a digital representation of the ground surface topography (terrain) provided in the form of a Digital Elevation Model (DEM). The main advantage of this approach is that the flow can be resolved very accurately at the boundaries of the variable terrain, which is essential in the case of the formation of shear layers along these solid boundaries. To generate a wind field library for the wind turbine farm and the surrounding environs, the CFD model urbanSTREAM was executed in a standalone mode (namely, the model was not coupled at this stage to the mesoscale model WRF). A power-law velocity profile with the following functional form was used to define the inflow boundary conditions for the simulations:

(12) $$\begin{array}{c} \frac{V\left(z\right)}{V_{10}}={\left(\frac{z}{10}\right)}^{s}, \end{array}$$

where *V*_10_ is the reference mean wind speed at 10 m height above ground level, *z* is the height in meters above the ground, and the exponent *s* depends on the nature of the surface terrain and the atmospheric stability. For the wind field library generation, simulations were carried out to provide dimensionless wind fields over the region occupied by the wind farm, with each of these wind fields being normalized by the reference wind speed *V*_10_. In these simulations, we also assumed that the gradient mean wind extended only to a height of about 400 m above ground level. Above this height, the flow was approximated as a shear-free (or frictionless) flow (namely, ∂*V*/∂*z* = 0).

In order to account for the effect of significant surface roughness on the wind speed, the drag-force model proposed by Lien et al. [29] was used. In this approach, we solved a time-averaged and spatially-averaged Navier-Stokes (NS) equation that involved the inclusion of an additional body force (drag force) *F*_*d*_ with the general form

(13) $$\begin{array}{c} F_{d}=-C_{D}\hat{A}\left[\left(Q+\frac{2}{3}\frac{k}{Q}\right)\langle \bar{u_{i}}\rangle -\nu_{t}\left(\frac{\partial \langle \bar{u_{i}}\rangle }{\partial x_{j}}+\frac{\partial \langle \bar{u_{j}}\rangle }{\partial x_{i}}\right)\right]. \end{array}$$

In (13), $Q\equiv (\langle \bar{u_{i}}\rangle \langle \bar{u_{i}}\rangle {)}^{1/2}$ is the spatially-averaged, time-mean wind speed; 〈*ϕ*〉 and $\bar{\phi }$ are used to denote the spatial (volume) and time average, respectively, of any flow variable *ϕ*; *ν*_*t*_ ≡ *C*_*μ*_ *k* ^2^/*ϵ* is the eddy (or turbulent) viscosity, where *C*_*μ*_ = 0.09 is a closure constant, *k* is the turbulent kinetic energy, and *ϵ* is the viscous dissipation; *C*_*D*_ and $\hat{A}$ are the drag coefficient and frontal area density of the surface roughness elements, respectively. The modeled transport equations for *k* and *ϵ* are given in Lien and Yee [30]. After some trial and error, it was found that the choice of *s* ≈ 0.12 (see (12)) and *C*_*D*_ ≈ 20 gave the best overall conformance of the CFD wind speed predictions with the measured wind speeds at the WT24 wind turbine location.

**Figure 1:**
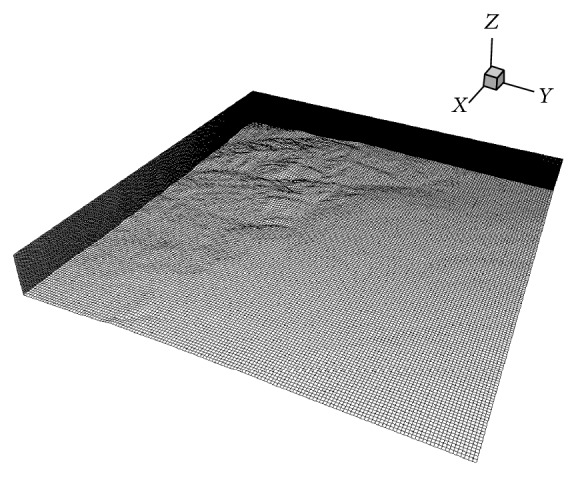
A 3D perspective view of the computational grid with 149 × 149 × 66 nodes.

For the high-resolution wind field predictions using CFD, a mesh of 149 × 149 × 66 grid lines in the Cartesian *x*-,*y*-, and *z*-directions, respectively, was used (see Figure 1) providing a horizontal spatial resolution of 85 m. A finer mesh of 301 × 301 × 66 grid lines was also employed (resulting in a horizontal spatial resolution of 42 m), but owing to the fact that the resulting surface topography was reasonably smooth, the coarser resolution grid results were deemed to be sufficient for the short-term wind power forecasting application considered herein.

The time-averaged and spatially-averaged NS equation (in the module urbanSTREAM) was solved numerically using a collocated, finite-volume method. The diffusive volume-face fluxes were discretized using a second-order accurate central differencing scheme, whereas the convective volume-face fluxes were approximated using a second-order accurate Upstream Monotonic Interpolation for Scalar Transport (UMIST) scheme [31]. The Semi-Implicit Method for Pressure-Linked Equations (SIMPLE) algorithm described in detail by Patankar and Spalding [32] was used to solve the pressure-correction equation. The continuity equation was enforced indirectly by solving this pressure-correction equation which, as part of the iterative sequence, steers the pressure towards a state in which all mass residuals in the grid cells are negligibly small. A nonlinear interpolation scheme [33] was used to interpolate the cell face velocities from the nodal velocities at the cell centers, in order to prevent checkerboard oscillations from developing in the pressure field. A wall function boundary condition was imposed on the mean velocity component tangential to the wall (solid surface) and the impermeability condition was imposed on the mean velocity component normal to the wall.

**Figure 2:**
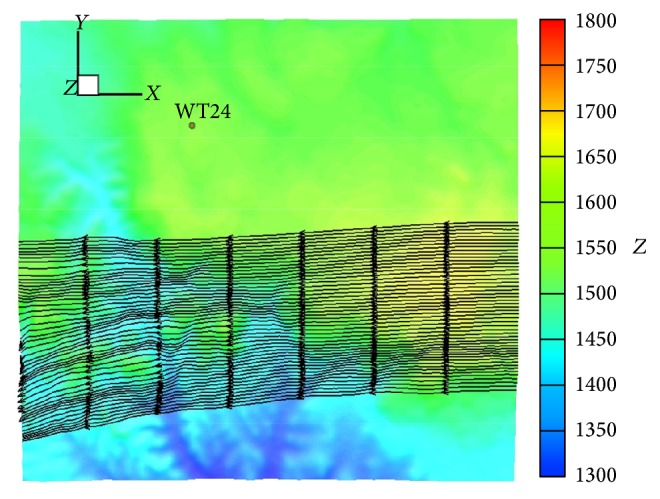
A top view showing the surface mean flow streamlines over the terrain occupied by the wind farm for an easterly incident wind direction. The terrain elevation above mean sea level (*Z*) in meters is color coded in the figure.

A precalculated wind field library was generated for the wind turbine farm and the surrounding environs using the high-resolution and high-fidelity CFD model urbanSTREAM. For the wind field library generation, simulations were carried out for 16 prevailing cardinal wind directions, with a wind direction interval of 22.5°. As an illustration of the wind flow patterns archived in the wind field library, Figure 2 depicts the mean flow streamlines near the ground surface at an incoming (incident) wind direction of 90° (namely, for an easterly incident wind). We have developed similar wind field libraries using urbanSTREAM in support of the 2010 Winter Olympic Games in Vancouver and of the 2010 Group of Eight (G8) and Group of Twenty (G20) Summits in Toronto [34]. To our knowledge, this was the first time that an operational demonstration was conducted showing the feasibility of using a building-aware wind field library at about 9 m resolution for a specific (predetermined) urban area to enable “on-demand” CFD predictions of urban dispersion for emergency response applications that required short turn-around times. Recently, Li et al. [6] also adopted a similar approach (which they referred to as “CFD precalculated flow fields”) to perform short-term wind speed forecasting.

Once the wind field library has been generated, it can be utilized as follows to provide a high-resolution prediction of the wind flow at any point in the computational domain (which includes the wind turbine farm). The information from the coarse-resolution (3 km) numerical weather prediction of the flow in the vicinity (obtained using the WRF model) was employed to unnormalize the flow fields in the wind field library, so that they can be used to provide a wind flow prediction at any point in the CFD spatial domain and at any time. Towards this purpose, if a prediction of the wind velocity is required at hub height at the location (*x*′, *y*′) of a particular wind turbine at time *t*, then the wind speed and direction predicted by the WRF model at the turbine hub height (65 m) at time *t* on the west, east, south, and north boundaries of the CFD flow domain were used firstly to unnormalize the various flow fields in the wind field library and subsequently to estimate (or predict) the required wind velocity using a weighted mean of these unnormalized wind fields.

As a first step, the wind speed and direction predicted by WRF at time *t* at the turbine hub height at the west, east, south, and north boundaries of the computational domain for the CFD simulations were used to unnormalize specific wind fields extracted from the library. For each of these boundaries, the WRF wind direction at the turbine hub height is used to select two wind field members from the library corresponding to the two incident (prevailing) wind directions that bracket the given WRF wind direction. Linear interpolation is applied to these two wind field members to obtain the normalized wind velocity at the location (*x*′, *y*′). Next, for each of the computational boundaries, the WRF wind speed at the turbine hub height was used to unnormalize the so-obtained wind velocity. More specifically, the WRF wind speed at the turbine hub height was used in relation to (12) to determine the reference wind speed *V*_10_ at 10 m height, which in turn can be used to unnormalize the wind velocity at (*x*′, *y*′). At this point in the process, there are four separate estimates for the unnormalized wind velocity at (*x*′, *y*′) at time *t* obtained from using the information embodied in the WRF wind speed and direction at the west, east, south, and north boundaries of the computational domain. The final step involves computing a weighted mean of these four wind velocity predictions (estimates) in order to give the prediction of the wind velocity at (*x*′, *y*′). The weights used in this average are the probabilities for observing incident (inflow) wind directions through the west, south, east, and north boundaries of the computational domain. For the purpose of determination of the probability weights, incident easterly, southerly, westerly, and northerly winds are assumed to correspond to incoming wind directions *θ* in the sector defined by *θ* ∈ (45°, 135°), (135°, 225°), (225°, 315°), and (−45°, 45°), respectively.

### 3.4. Forecast Analysis

**Figure 3:**
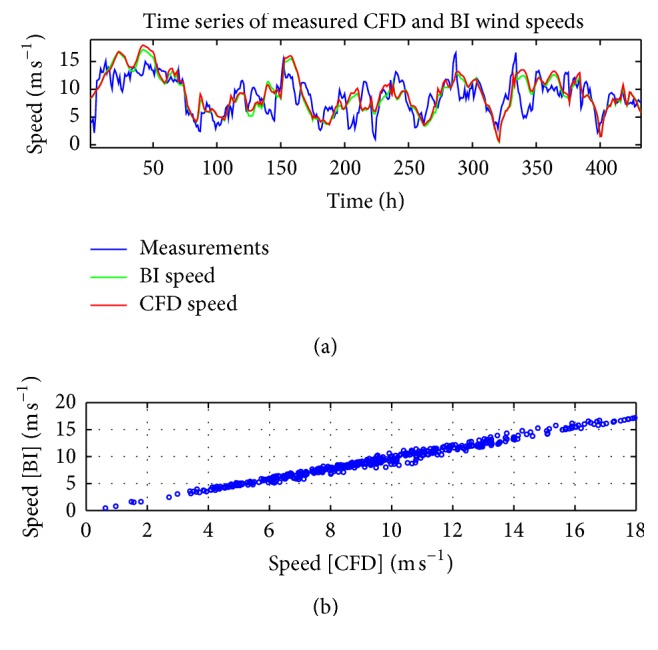
(a) Comparison between the measured wind speeds and the model wind speeds obtained from BI of NWP data and from the CFD wind field library for wind turbine number 24 (WT24). (b) The correlation between the model wind speeds obtained from BI of NWP data and from the CFD wind field library.

Before we train the ANNs and use them for forecasting, let us first examine the model wind speeds obtained from the CFD wind field library and NWP database for WT24. The two time series of these model wind speeds are shown in Figure 3, together with the measured wind speed at WT24. It is seen that the model wind speed time series obtained from CFD or from BI of NWP data are very similar and indeed the correlation between the CFD and BI wind speeds is seen to be high (cf.  Figure 3(b)). Furthermore, both of these model wind speeds are seen to exhibit discrepancies with the associated measured wind speeds, although the model and measured wind speeds are observed to follow the same general (large-scale) trends.

Firstly, we conducted an empirical analysis of wind speed forecasting using multiple ANNs, each of which is trained using the measured historical wind speed data and model wind speed data obtained either from the CFD wind field library or from the BI of the NWP data. To reduce the effects of the uncertainty in the ANN model structure for forecasting, we trained an ensemble of three-layer ANNs with member neural networks having various numbers of nodes in the hidden layer, ranging from 5 to 30 inclusive (namely, the ensemble consists of 26 different model structures for ANNs, each structure differing in the number of hidden units used to represent the training data). Furthermore, in order to reduce the uncertainty arising from the initialization of the weight vector for the ANN, for each of the 26 ANN model structures in the ensemble five randomly selected weight vectors were used for training the ANN. In consequence, the ensemble of ANNs consists of 130 members (namely, 26 different model structures, each of which is trained five times starting from five different weight initializations). Each of the trained ANNs in the ensemble can be used to provide a multi-step-ahead wind speed forecast, with the ensemble aggregate forecast obtained by weighted averaging of all of the forecasted wind speeds from the ensemble members (with weights determined through the solution of a quadratic programming problem described above). This model-averaged forecast over an ensemble of ANNs overcomes the problem of choosing the number of hidden layer nodes to use and avoids the problem of overfitting and poor generalization associated with choosing too many nodes and too few nodes, respectively. We used the PSO methodology for neural network training for each member of the ensemble. The multi-step-ahead wind speed and wind power forecasts can be obtained by averaging the predictions from all the ensemble members. The confidence interval for this ensemble-averaged prediction can be obtained by using a Student's *t*-distribution with *v* = 129 degrees of freedom (since there are 130 independent forecasts of the wind speed or power at each time point).

**Figure 4:**
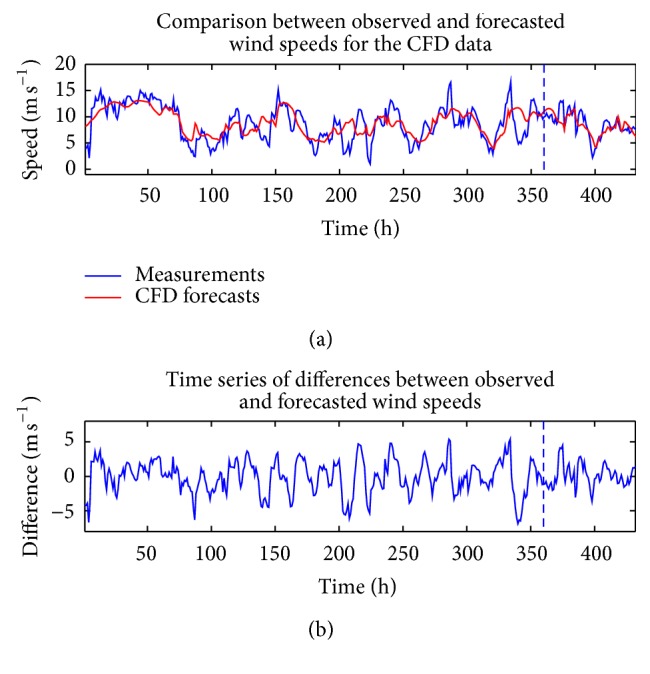
(a) In-sample “predictions” and out-of-sample multi-step-ahead forecasts of wind speeds obtained from the best-fitting ANN which used the CFD wind speed data as the exogenous input for a NARX(0,0) ANN. (b) The discrepancy between the measured and predicted wind speeds.

**Figure 5:**
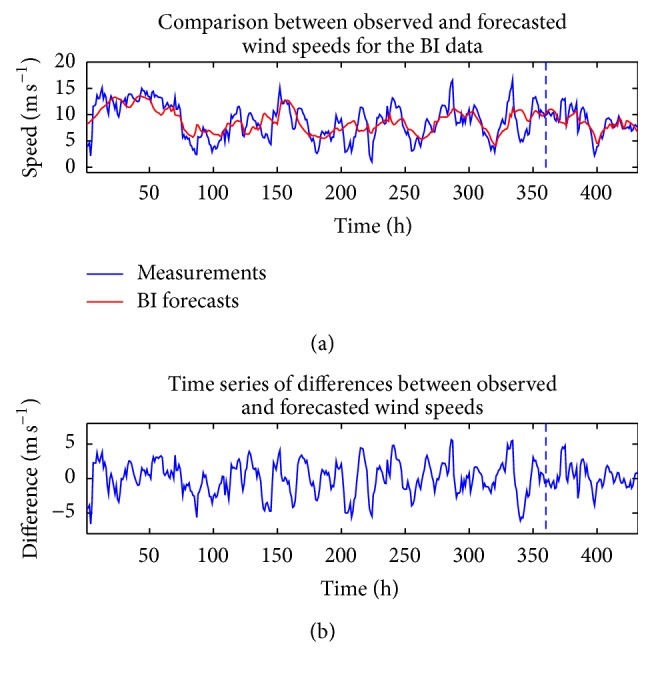
(a) In-sample “predictions” and out-of-sample multi-step-ahead forecasts of wind speed obtained from the best-fitting ANN which used the BI wind speed data as the exogenous input for a NARX(0,0) ANN. (b) The discrepancy between the measured and predicted wind speeds.

Figures 4 and 5 compare the measured wind speeds at WT24 with the in-sample “predictions” (these so-called in-sample “predictions” are more precisely described as retrodictions since these so-called “predictions” gauge how well the model fits the data used to train the ANNs) and the out-of-sample multi-step-ahead forecasts of the wind speed for a single (individual) ANN which used the CFD and BI wind speed data as the exogenous inputs, respectively. The forecasts in these figures used the NARX(0,0) ANN model. Furthermore, these forecasts correspond to the individual ANN that yielded the best fit to the training data. The data is split into two groups, which are delineated by the vertical dashed line in these figures. The data to the left of the vertical dashed line (approximately 83% of the total data) were used for training the network and those to the right of the line (approximately 17% of the total data) were used as validation data to assess the prediction efficacy.

**Figure 6:**
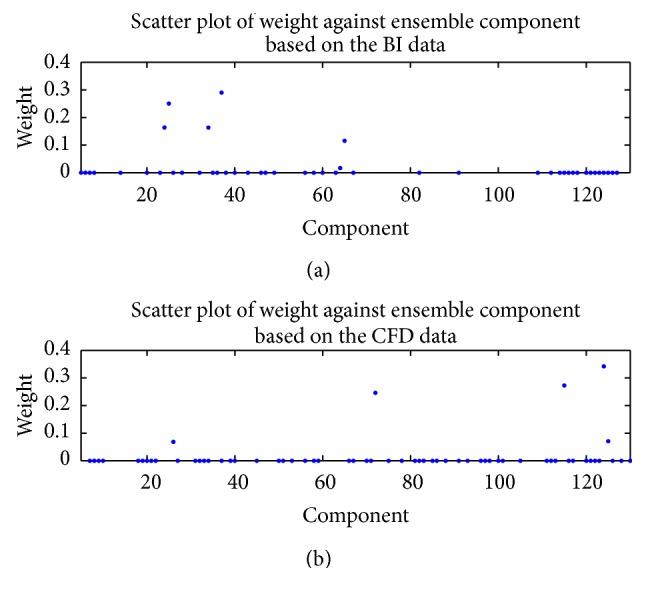
Plot of the nonzero weights against the corresponding ensemble components for the ensemble of NARX(0,0) ANNs that used the BI wind speed data (a) and the CFD wind speed data (b) for the exogenous input.

Next, we consider in-sample and out-of-sample predictions of the wind speed using the ensemble of ANNs for the NARX(0,0) ANN model. For this purpose, Figure 6 displays a plot of the nonzero weights (used in the weighted averaging of the members of the ensemble of ANNs) against the corresponding ensemble components (members) for the cases when the BI wind speed data (a) and the CFD wind speed data (b) were used as the exogenous inputs, respectively. Note that only 37% of the members in the ensemble based on the BI wind speed data and only 46% of the members in the ensemble based on the CFD wind speed data contributed nonzero weights to the aggregate (ensemble) predictions.

**Figure 7:**
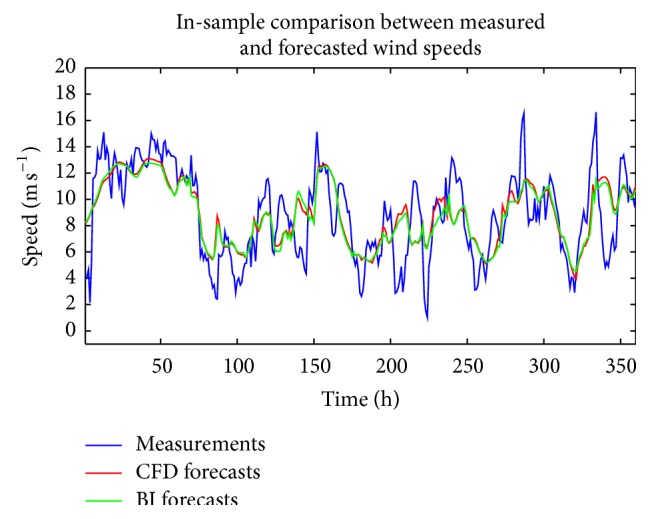
In-sample ensemble wind speed “predictions” obtained from weighted averaging of ANN “predictions” from ensemble members using CFD and BI wind speed data as the exogenous input for NARX(0,0) ANNs.

**Figure 8:**
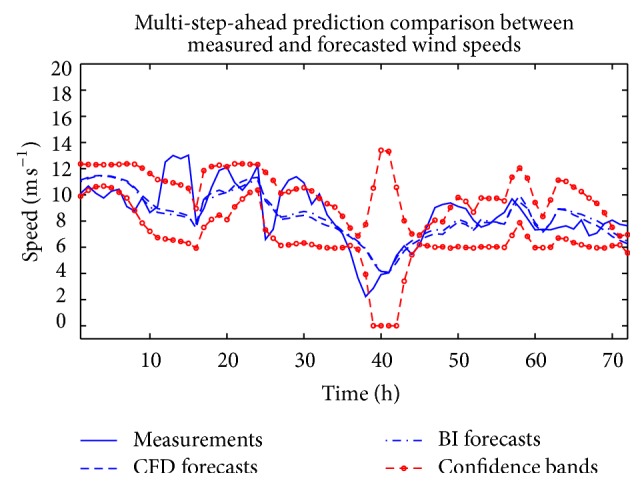
Out-of-sample multi-step-ahead ensemble wind speed forecasts obtained from weighted averaging of ANN forecasts from ensemble members using CFD and BI wind speed data as the exogenous input for NARX(0,0) ANNs. The dashed lines marked with circles delineate the 95% confidence intervals for the ensemble wind speed prediction using the CFD data.

Figure 7 displays the in-sample aggregate “predicted” wind speeds obtained from the ensemble of ANN models trained with the CFD and BI wind speed data, whereas Figure 8 shows the multi-step-ahead ensemble forecasted wind speeds. These forecasts were obtained from various NARX(0,0) ANNs that constituted the members of the ensemble. In both of these figures, the ensemble forecasts of the wind speed are compared to the corresponding measured wind speeds (at hub height for WT24). In Figure 8, the dashed lines marked with circles delineate the 95% confidence interval for the ensemble predicted wind speeds obtained using the CFD wind speed data (as the exogenous input for the ANN). Finally, Table 2 summarizes the values of RMSE and MAE for various cases involving the use of CFD and BI wind speed data as exogenous inputs for NARX(0,0) ANNs. These cases include the values associated with the ANN providing the best forecast in the ensemble, as well as those associated with the ensemble forecast. In addition, the values for RMSE and MAE resulting from using a persistence forecast (a persistence forecast uses the current wind speed to predict the value of the future wind speed) are also included in the table.

A perusal of the table indicates that the ensemble forecast using the BI of NWP data as the exogenous input provides the best estimate for the future wind speeds as measured using MAE. Indeed, with respect to MAE, it is seen that the weighted average of ensemble ANN members provides generally better predictions for the future wind speeds than the single best forecast member when using either CFD or BI of NWP data as exogenous inputs. However, when prediction errors are determined using RMSE, the opposite tendency appears to hold; namely, the single best forecast member using either CFD or BI of NWP data as exogenous inputs tends to provide better predictions of future wind speeds than the aggregate forecast using a weighted mean of the ensemble members. However, the differences in the prediction performance as gauged by the RMSE between the best individual and weighted ensemble-mean forecasts for these cases are small.

**Figure 9:**
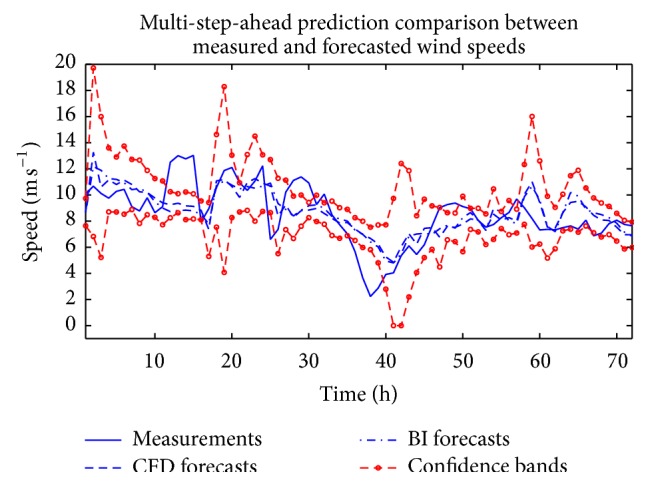
Out-of-sample multi-step-ahead ensemble wind speed predictions obtained from weighted averaging of ANN forecasts from ensemble members using CFD and BI wind speed data as the exogenous input for NARX(*p*, *q*) ANNs (with *p* > 0 and *q* > 0). The dashed lines marked with circles delineate the 95% confidence intervals for the ensemble wind speed prediction using the CFD data.

The analysis described above was repeated with NARX(*p*, *q*) ANNs with *p* ∈ {1,2} and *q* ∈ {1,2} in order to investigate whether the inclusion of temporal correlation in the dependent and exogenous variables would lead to better forecasts for the wind speed. The combination of *p* and *q* with *p* ∈ {1,2} and *q* ∈ {1,2} that yielded the best performance on the training set was selected as a component (member) ANN in the ensemble. The weighted average of the component ANNs thus obtained was used to provide a short-term forecast of the wind speed. The results of this forecast are displayed in Figure 9 for two cases: for the case when the CFD wind data was used as the exogenous input and for the case when the BI of the NWP data was used as the exogenous input for the NARX(*p*, *q*) ANN model (with *p* > 0 and *q* > 0 to incorporate the effects of correlation in the dependent and exogenous variables in the prediction). In addition, the 95% confidence intervals for this prediction using the CFD wind data as the exogenous input are also exhibited in the figure. The forecast figures of merit are summarized in Table 3. A perusal of this table indicates that, for both the RMSE and MAE, the forecast obtained from the weighted ensemble average using the CFD wind data as the exogenous input yields the best prediction performance.

A comparison of Tables 2 and 3 shows that the NARX(0,0) ANNs provide a better forecast of the wind speed than the NARX(*p*, *q*) ANNs (with *p* > 0 and *q* > 0), suggesting that in this case there is no information in the correlation structure of the hourly-averaged wind speeds (either observed or modeled) that can be exploited to provide a better prediction. Indeed, it is seen that inclusion of the correlation leads to ANNs that provide poorer predictions in general. This perhaps is not so surprising. For hourly-averaged wind speed data (either observed or modeled), the temporal correlations in the wind speed arising from the small-scale action of surface friction (wind shear) and buoyancy forces in the atmospheric boundary layer are removed by the temporal averaging (1 h) of the wind speed. Of course, there may be correlations that exist on longer time scales than 1 h, but the presence of the spectral “gap” in the wind speed spectrum [35] implies that there is absence of physical mechanisms for the production of large wind fluctuations on scales intermediate between the smaller-scale turbulent scales and the larger scale action of the earth's rotation. As a consequence, for hourly-averaged wind speeds, the exclusion of temporal correlation in the modeling leads to ANNs with better generalization capabilities (by the avoidance of model “overfitting”).

**Figure 10:**
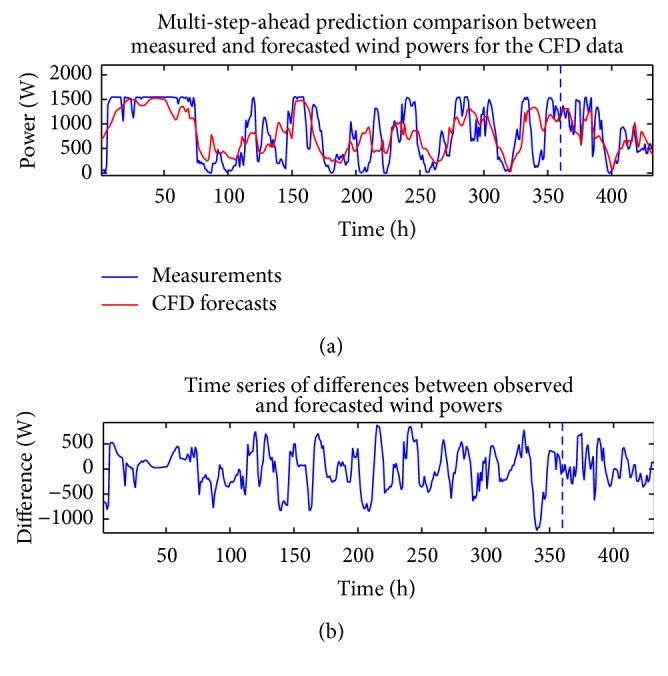
In-sample “predictions” and out-of-sample multi-step-ahead forecasts of the wind power for the best-fitting ANN obtained from using the CFD wind speed data as the exogenous input (a) and discrepancies between the forecasted and measured wind power (b). These predictions were obtained with a NARX(0,0) ANN model structure.

**Figure 11:**
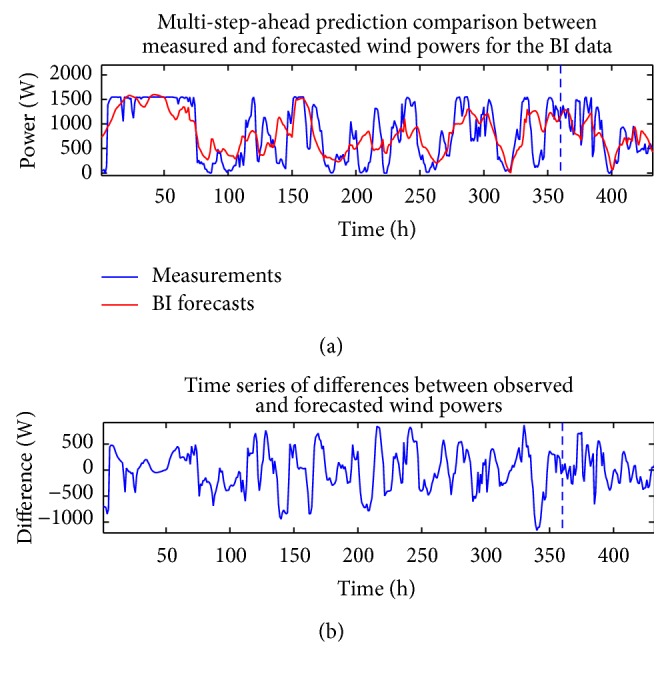
In-sample “predictions” and out-of-sample multi-step-ahead forecasts of the wind power for the best-fitting ANN obtained using the BI wind speed data as the exogenous input (a) and discrepancies between the forecasted and measured wind power (b). These predictions were obtained with a NARX(0,0) ANN model structure.

**Figure 12:**
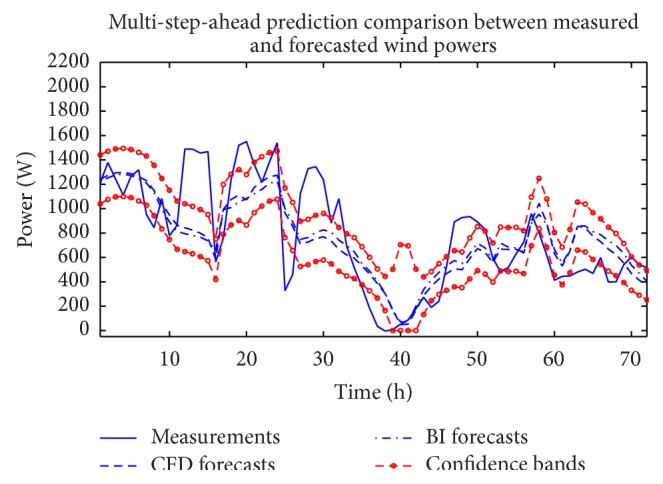
Out-of-sample multi-step-ahead ensemble forecasts of the wind power for the cases when the CFD and BI wind speed data were used as exogenous inputs. The dashed lines marked with circles delineate the 95% confidence intervals for the ensemble wind power prediction using the CFD data. The predictions were obtained using a NARX(0,0) ANN model structure.

The analysis described above in this section for the wind speed can also be applied to the measured wind power time series and the CFD and BI model wind speed data (exogenous variables). Figures 10 and 11 compare the measured wind power with the in-sample “predictions” and out-of-sample multi-step-ahead forecasts of the wind power using the single best-fitting ANN prediction for the cases when the CFD and BI wind speed data were used as exogenous inputs, respectively. These predictions are obtained with a NARX(0,0) ANN model structure.  Figure 12 shows the associated multi-step-ahead ensemble forecast of the wind power for the CFD and BI wind speed data, along with the 95% confidence intervals for the predicted wind power obtained using the CFD wind speed data as the exogenous input. These ensemble forecasts are compared with the measured wind power for WT24. Finally, Table 4 summarizes the values of RMSE and MAE for the single best-fitting ANN as well as for the ensemble ANN for the cases when the CFD and BI wind speed data were used as the exogenous variables in the prediction. For reference, the values of RMSE and MAE for a simple persistence forecast are also included in the table. An examination of the table indicates that the ensemble ANN using the CFD wind speed data as the exogenous input provides the best predictions for the wind power with respect to both the RMSE and the MAE criteria (albeit the prediction is only slightly better than that provided by the ensemble forecast which used the BI wind speed data as the exogenous input).

**Figure 13:**
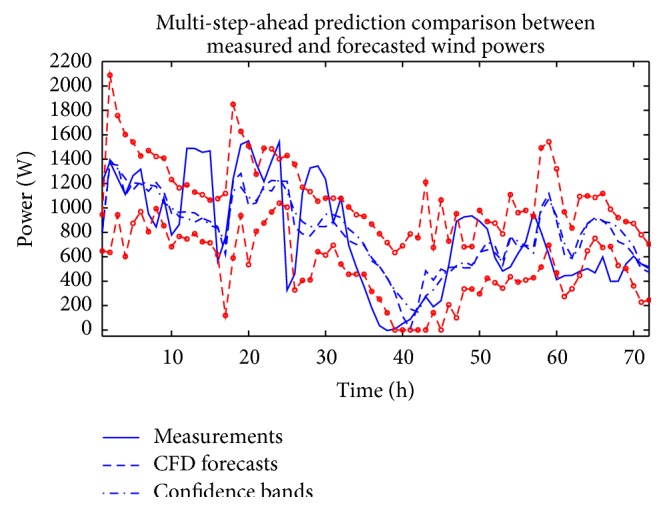
Out-of-sample wind power prediction obtained from a NARX(*p*, *q*) ANN ensemble with *p* > 0 and *q* > 0.

As with the wind speed, we used also a NARX(*p*, *q*) ANN model structure (*p* > 0 and *q* > 0) to provide an ensemble of ANNs for wind power forecasting.  Figure 13 presents the results of out-of-sample weighted ensemble-averaged short-term wind power forecasts using this more complicated model structure that includes correlation in both the dependent (wind power) and exogenous (CFD or BI of NWP wind data) variables. The ensemble-averaged wind power forecasts shown here include predictions obtained using the CFD and BI of NWP wind data as exogenous variables. In addition, the 95% confidence intervals for the predictions derived from the ensemble that used the CFD wind data as the exogenous input for the NARX(*p*, *q*) ANN model are also displayed in the figure.  Table 5 summarizes the prediction performance of the NARX(*p*, *q*) ANN model ensemble in terms of the RMSE and MAE. A comparison of the results in this table with those in Table 4 shows that the inclusion of correlation in the dependent and exogenous variables degrades the prediction performance (something that is already expected from the results for the wind speed forecasting reported above).

## 4. Empirical Analysis of the Censored Wind Power Prediction

**Figure 14:**
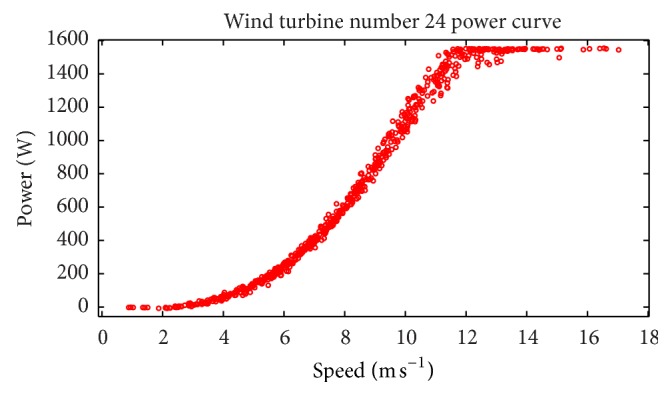
Measured power curve for WT24.

Next, we show how to take into account the fact that the wind power is censored from above. It is important to note that a wind turbine has a maximum capacity for the generation of wind power. In other words, when the wind speeds exceed a certain value (the so-called rated output wind speed), a limit to the turbine power generation is attained and for wind speeds which exceed the rated output wind speed, the wind turbine is designed to restrict the power output to this (maximum) limit. Consequently, the power generated by a wind turbine is necessarily censored from the above. To account for this maximum upper bound for the wind power generation, the CFD and BI model wind speed data (exogenous variables) were adjusted to match the censored wind power before they were used for the neural network training. More specifically, if the CFD and BI model wind speeds exceeded 11.5 m s^−1^ (rated output wind speed for WT24), then the wind power associated with this range of wind speeds was limited to 1550 W (rated output power for WT24).  Figure 14 exhibits the measured wind turbine power output as a function of the measured wind speed (power curve) for WT24.

**Figure 15:**
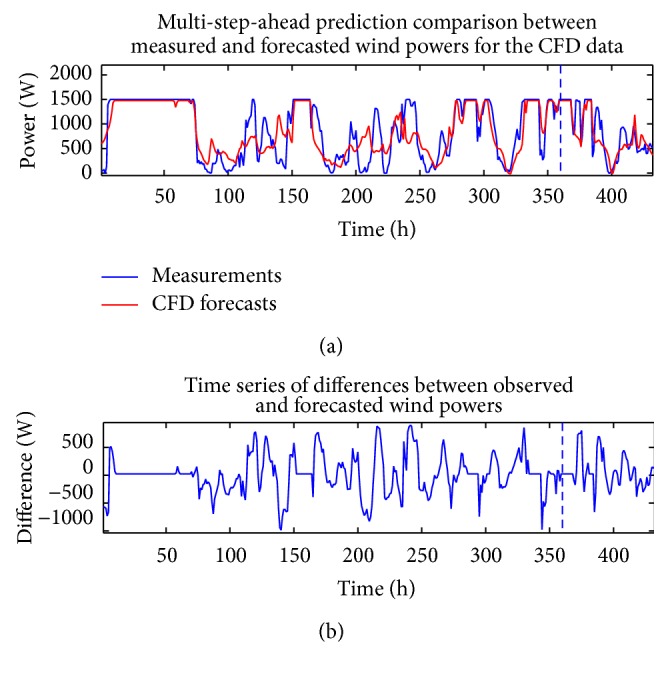
(a) In-sample “predictions” and out-of-sample multi-step-ahead censored wind power prediction using the best-fitting ANN trained with the CFD wind speed data as the exogenous input. (b) The discrepancy between the predicted and measured wind power.

**Figure 16:**
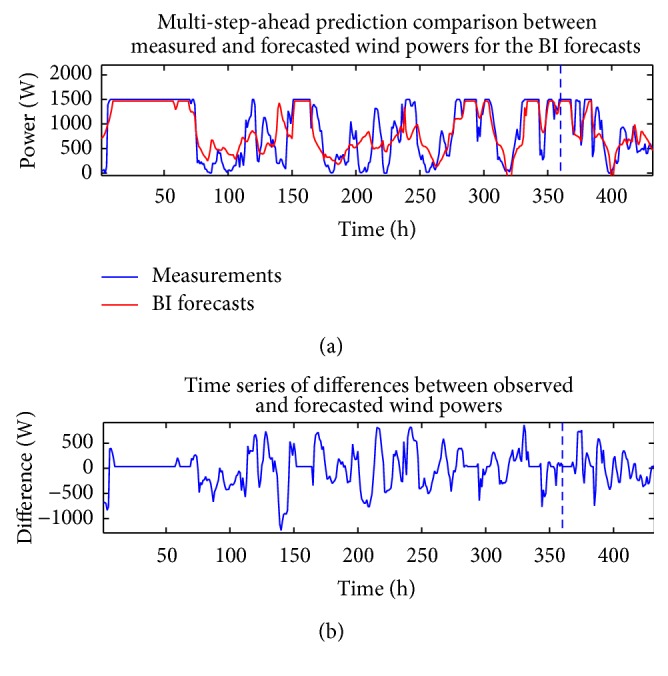
(a) In-sample “predictions” and out-of-sample multi-step-ahead censored wind power prediction using the best-fitting ANN trained with the BI wind speed data as the exogenous input. (b) The discrepancy between the predicted and measured wind power.

**Figure 17:**
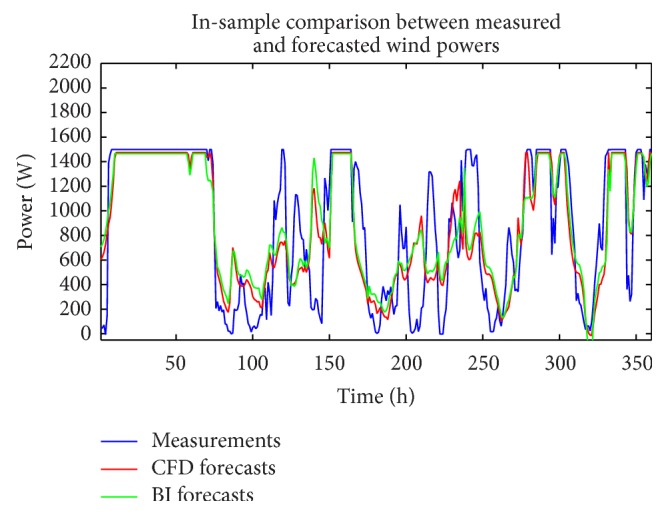
In-sample weighted ensemble-averaged censored wind power prediction for the CFD and BI wind speed data.

**Figure 18:**
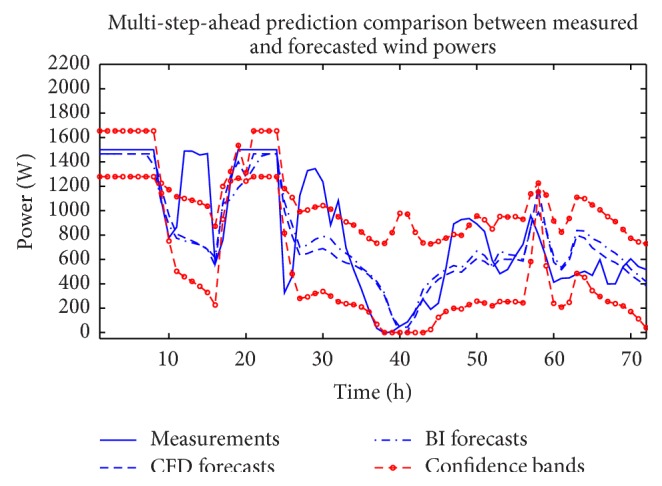
Out-of-sample multi-step-ahead weighted ensemble-averaged censored wind power prediction for the CFD and BI wind speed data. The dotted-dashed lines delineate the 95% confidence intervals for the wind power prediction obtained using the CFD wind speed data.

Using the adjusted CFD and BI wind speed data as exogenous inputs to an ANN, we trained an ensemble of neural networks for modeling the wind power output (accounting properly for the fact that this quantity is censored from above). The NARX(0,0) ANN model structure was used for this analysis (as it was shown above that the inclusion of temporal correlation in the model resulted in poorer prediction performance). Figures 15 and 16 compare the measured wind power at WT24 with the in-sample “predictions” and the out-of-sample multi-step-ahead forecasts of the wind power for, respectively, the CFD and BI wind data exogenous inputs. In each case, these forecasts were obtained from the single ANN which provided the best fit to the training data. Along the same vein, Figures 17 and 18 compare the measured wind power with the in-sample “predictions” and out-of-sample multi-step-ahead weighted ensemble-average forecasts of the wind power obtained using the CFD and BI wind data. Furthermore, the 95% confidence intervals for the predicted wind power are superimposed on Figure 18 for the CFD wind data. Finally, Table 6 provides the values of RMSE and MAE for the various ANN predictions which can be compared with the simple persistence forecast (reference). It is observed that the ensemble ANN that uses the CFD wind speed data as the exogenous input provides a better model for wind power prediction in accordance with the MAE criterion. For the RMSE criterion, the ensemble ANN that employs the BI wind speed data as exogenous input gives the best performance for power forecasting, although this performance is only marginally better than that provided by the CFD wind speed data. A comparison of the RMSE and MAE values for comparable cases in Tables 4 and 6 shows that accounting properly for the wind power censoring in the ANN training resulted in better forecasts for the wind power (as determined by the smaller misfits between the forecasted and measured wind power when the censoring was applied, at least as measured by the MAE, which is a more robust measure for misfit relative to the presence of outliers in the data).

## 5. Conclusions

In this paper, we proposed an ensemble ANN model for wind speed and wind power forecasting based on a NARX(*p*, *q*) model structure. The examples considered herein suggest that combining forecasts for wind speed and power from the ensemble ANN members using a weighted averaging scheme generally provided predictions with improved forecast accuracy compared to those obtained from a single best set of ANN parameters. Furthermore, using an ensemble ANN framework has the additional benefit of providing a measure of the degree of uncertainty in the forecasts in the form of confidence intervals at a specified probability level. It has been shown that the uncertainty bounds defined at the 95% confidence interval for the wind speed and power forecasting example considered in this paper are consistent with the statistical properties of the wind turbine observations of speed and power and would support generally the validity of the approach for performing accurate probabilistic wind speed and power forecasting.

For hourly-averaged observed and modeled wind speeds (the latter used as exogenous inputs for the ANN), the NARX(0,0) model structure (which excludes the use of temporal correlation information in the dependent and exogenous variables in the ANN) gave the best short-term forecasts for both the wind speed and power. The inclusion of temporal correlation in the dependent and exogenous variables by using a NARX(*p*, *q*) model with *p* > 0 and *q* > 0 (when such a correlation is absent) led generally to overfitting and poor generalization for the neutral network compared to the forecast performance of the NARX(0,0) model structure.

We also proposed to use the precalculated wind speed data from a wind field library constructed using a CFD model as the exogenous input for neural network training. It was found that the CFD wind speed exogenous input generally yielded more accurate forecasts for the wind speed and power than those that were obtained from a simple bilinear interpolation of the NWP wind speed data. For wind turbine 24 used in this study, the improvement in forecast accuracy using CFD wind data rather than a bilinear interpolation of (much) coarser-resolution NWP data was marginal to moderate owing to the fact that this turbine was situated on open and flat terrain. However, for wind turbines located on more complex terrain, it is expected that using the higher-resolution CFD wind data as the exogenous input for ANNs would have resulted in significantly better forecast performance when compared to that using the BI of the coarser-resolution NWP wind data which did not adequately resolve the detailed features of the surrounding terrain. Finally, we showed that the ANN methodology can be applied to censored wind power data and that when the censoring is accounted for properly in the ANN training, the ability of the network to provide accurate forecasts for the wind power improved.

## Figures and Tables

**Table 1:** Activation functions used in ANNs.

Function name	Transfer function *F*
Linear	*F*(*x*) = *x*
Hyperbolic tangent sigmoid	$F\left(x\right)=\frac{e^{x}-e^{-x}}{e^{x}+e^{-x}}$
Logistic sigmoid	$F\left(x\right)=\frac{1}{1+e^{-x}}$
Gaussian radial basis function	$F(x)=\exp\left(-\frac{1}{2\sigma^{2}}{||x-\overset{-}{x}||}^{2}\right)$

**Table 2:** Wind speed forecast assessment for various cases using NARX(0,0) ANNs.

Criterion	Persistence	BI best	CFD best	BI average	CFD average
RMSE	2.3554	1.6532	1.6453	1.6592	1.6620
MAE	1.8693	1.2484	1.2756	**1.2386**	1.2717

**Table 3:** Wind speed forecast assessment for various cases using NARX(*p*, *q*) ANNs with *p* > 0 and *q* > 0.

Criterion	Persistence	BI best	BI average	CFD best	CFD average
RMSE	2.3554	1.7679	1.7895	1.8138	1.7285
MAE	1.8693	1.3701	1.3589	1.4317	1.3411

**Table 4:** Wind power forecast assessment for various cases using NARX(0,0) ANNs.

Criterion	Persistence	BI best	CFD best	BI average	CFD average
RMSE	499.7206	302.2296	297.6311	300.8724	**295.9451**
MAE	429.0748	238.3083	231.3721	235.7355	**230.3702**

**Table 5:** Wind power forecast assessment for various cases using NARX(*p*, *q*) ANNs with *p* > 0 and *q* > 0.

Criterion	Persistence	BI best	BI average	CFD best	CFD average
RMSE	499.7206	321.8786	**317.9381**	331.2846	321.2846
MAE	429.0748	261.5242	261.0886	272.4298	**260.4414**

**Table 6:** Forecast assessment of the censored wind power.

Criterion	Persistence	BI best	CFD best	BI average	CFD average
RMSE	811.652	297.9009	302.6383	** 297.4883 **	299.1062
MAE	653.2516	223.6501	213.8122	219.2275	**212.8934**
